# Post-thyroidectomy Depression and Associated Factors in Saudi Arabia

**DOI:** 10.7759/cureus.55328

**Published:** 2024-03-01

**Authors:** Shaima A Althoubaiti, Amirah S Alharthi, Reham M Al Kahtani, Mayar A Algrni, Amal G Alshorm, Mohammad Eid M Mahfouz

**Affiliations:** 1 Department of Surgery, College of Medicine, Taif University, Taif, SAU

**Keywords:** saudi population, postoperative, phq9, depression, thyroidectomy

## Abstract

Background

Thyroidectomy is a common surgical procedure used to treat thyroid gland illnesses. The surgery has many outcomes, and one of them may show an association with depression. This study aims to assess the factors associated with depression after thyroidectomy in Saudi Arabia.

Methodology

A cross-sectional study was conducted among 414 Saudi participants. The target population included patients more than 18 years old and who had undergone thyroidectomy, whereas patients 18 years or younger were excluded. The data were collected between December 2023 and January 2024 using an electronic self-administered questionnaire that included demographics, clinical characteristics, and the nine-item Patient Health Questionnaire. The questionnaire was distributed randomly throughout social media, and patient consent was obtained. The descriptive and inferential analyses were performed using SPSS software version 27 (IBM Corp., Armonk, NY, USA).

Results

The study showed that of the 414 participants, 306 were females and 108 were males. Depression affected 335 (80.92%) participants and was mostly mild (120, 28.99%), followed by moderate (109, 26.33%), moderately severe (55, 13.29%), and severe (51, 12.32%). Depression symptoms were more common in females than males. The participants who underwent total thyroidectomy (217, 52.41%) were more than those who underwent partial thyroidectomy (197, 47.58%). Temporary complications were more prevalent in the participants exhibiting symptoms of depression. Both educational level and surgery time were significant factors.

Conclusions

The study revealed a significant prevalence of post-thyroidectomy depression. The associated factors in post-thyroidectomy depression included educational level, with more depression symptoms noted with high education. In addition, surgery time showed an increased risk of developing depression that still existed two years postoperatively.

## Introduction

Thyroidectomy is a well-known procedure that removes the whole thyroid gland or a part of the gland in cases of thyroid illness that do not respond to medical treatment [1]. The thyroid gland has the following three components: the isthmus, which connects the two lobes below the cricoid cartilage; and the right and left lobes, which are located anterolateral to the larynx and trachea [2]. Therefore, thyroidectomy has many types, namely, total thyroidectomy (removal of all thyroid tissue), lobectomy (removing an entire lobe), and subtotal thyroidectomy (removing the gland and leaving >1 g of thyroid gland tissue) [1]. Nowadays, total thyroidectomy is the most common surgical procedure used to treat thyroid conditions [3].

Thyroidectomy can be indicated in both benign and malignant conditions such as thyroid cancer, thyroid nodules, Graves’ disease, and goiter with compression symptoms [4]. The complications of thyroidectomy can be transient, such as hypocalcemia, hoarseness, hematoma, and infection, or can be permanent, such as hypothyroidism, hypoparathyroidism, and unilateral vocal cord paralysis [5]. Hypocalcemia is a common and serious complication of total thyroidectomy, whereas hypothyroidism is seen as an expected outcome [6,7].

The outcome of thyroidectomy can be influenced by the type of surgery performed and the surgeon’s experience, with a more skilled surgeon linked to a better outcome, and total thyroid removal is associated with a higher risk of complication [8,9]. The quality of life after a thyroidectomy significantly improves compared to before the procedure [10]. According to statistics, patients may experience some psychological symptoms, such as depression, anxiety, and other issues, that could negatively impact their quality of life, especially within the first year after surgery [11,12].

Due to limited data in this field, in this study, we aim to highlight the assessment of the factors associated with depression after thyroidectomy in Saudi Arabia.

## Materials and methods

Study design, setting, and participants

This observational, cross-sectional study was conducted among 414 people to assess the prevalence of depression after thyroidectomy and the associated factors in the Saudi Arabian population from December 2023 to January 2024. The target population included patients aged more than 18 years who had undergone thyroidectomy, either or total removal of the gland, whereas patients 18 years of age or younger who had not undergone thyroidectomy were excluded.

Sample size

We calculated the sample size using the following formula: n = z^2^ p(1-p)/M^2^, where n is the number of samples, z is the level of confidence (95%), P is the population proportion (50%), and M is the margin of error (0.05). The calculated sample was 384, but we increased the sample size to 414, for more representation and to improve the accuracy and reliability of the study findings.

Data collection

An electronic, self-administered Arabic online questionnaire was developed using Google Forms. To ensure a diverse representation of participants from various regions, the questionnaire was randomly distributed through social media platforms such as Telegram and WhatsApp. The questionnaire was developed after conducting extensive literature reviews by researchers and doctor consultations and comprised the following three parts: the first part contained informed consent and demographic data (age, gender, region, educational level, occupation); the second focused on clinical factors (family history of depression, type of disease, type of surgery, time of surgery, complications, duration of complications); and the third part was a self-administered depression assessment scale known as the nine-item Patient Health Questionnaire (PHQ-9), which consists of nine questions designed to screen for depression symptoms. Each question is scored from 0 (not at all) to 3 (nearly every day), with a minimum score of 0 to a maximum score of 27; a higher score indicates more severe symptoms. We used the Arabic version [13].

Data analysis

The data were entered into Microsoft Excel 2019 (Microsoft Corp., Redmond, WA, USA), and then the data were analyzed using SPSS software version 27(IBM Corp., Armonk, NY, USA). Frequency and percentages were used to demonstrate categorical variables. Continuous variables were presented using the mean and standard deviation. To test for the presence of an association between categorical variables, a chi-square test was utilized, and the statistical significance level was set at 5%.

Ethical considerations

Ethical approval was obtained from the Research Ethics Committee of Taif University (application number: 45-087). Consent was obtained electronically from all participants after we explained the study’s aim. Confidentiality measures such as strict access to researchers only and anonymity were implemented for all collected data to ensure the privacy and confidentiality of participants’ information.

## Results

Demographic characteristics

A total of 414 participants were included in this study. Regarding age, most participants (48.31%) were between the ages of 19 and 30 years. Regarding gender, 73.91% of participants were female, while 26.09% were male. The participants were from various regions, with the highest percentage (34.54%) from the Western region and the lowest percentage (8.21%) from the North region. For educational level, the majority of participants (55.07%) had a university education. Regarding the area of work, the largest group (70.05%) consisted of participants who worked in various other fields not specified (Table 1).

**Table 1:** Demographic characteristics of the study participants (N = 414).

Characteristics	Categories	Frequency (n)	Percent (%)
Age (year)	19–30	200	48.31%
	31–50	167	40.34%
	Above 50	47	11.35%
Gender	Female	306	73.91%
	Male	108	26.09%
Regions	Western	143	34.54%
	Eastern	74	17.87%
	South	56	13.53%
	North	34	8.21%
	Central	107	25.85%
Education	Less than high school education	41	9.90%
	High school education	83	20.05%
	University education	228	55.07%
	Postgraduate	62	14.98%
Area of work	Broker for securities and commodities	10	2.42%
	Environmental management and waste service	2	0.48%
	Legal services	22	5.31%
	Manufacturing and production	11	2.66%
	Organization and association department	10	2.42%
	Otherwise	290	70.05%
	Personal services	11	2.66%
	Public or private transportation	16	3.86%
	Social service	21	5.07%
	Real estate	21	5.07%

Patient health survey

Table 2 shows the detailed assessment of participants’ depression based on the PHQ-9. Some of the most common items were feeling tired or having little energy (83.81%), feeling down or hopeless (79.71), having trouble falling or staying asleep, or sleeping too much (78.74).

**Table 2:** Assessment of depression after thyroid surgery based on the nine-item Patient Health Questionnaire (N = 414).

Symptoms	Not at all, n (%)	Several days, n (%)	More than half the day, n (%)	Nearly every day, n (%)
Little interest or pleasure in doing things	117 (28.26%)	159 (38.41%)	74 (17.87%)	64 (15.46%)
Feeling down, depressed, or hopeless	84 (20.29%)	185 (44.69%)	92 (22.22%)	53 (12.80%)
Trouble falling or staying asleep, or sleeping too much	88 (21.26%)	131 (31.64%)	106 (25.60%)	89 (21.50%)
Feeling tired or having little energy	70 (16.91%)	126 (30.43%)	114 (27.54%)	104 (25.12)
Poor appetite or overeating	109 (26.33%)	140 (33.82%)	81 (19.57%)	84 (20.29%)
Feeling bad about yourself—or that you are a failure or have let yourself or your family down	164 (39.61)	120 (28.99%)	59 (14.25%)	71 (17.15%)
Trouble concentrating on things, such as reading the newspaper or watching television	144 (34.78%)	129 (31.16%)	74 (17.87%)	67 (16.18%)
Moving or speaking so slowly that other people could have noticed or the opposite—being so fidgety or restless that you have been moving around a lot more than usual	196 (47.34%)	111 (26.81%)	58 (14.01%)	49 (11.84%)
Thoughts that you would be better off dead or of hurting yourself in some way	244 (58.94%)	80 (19.32%)	54 (13.04%)	36 (8.70%)

Prevalence of depression

**Figure 1:**
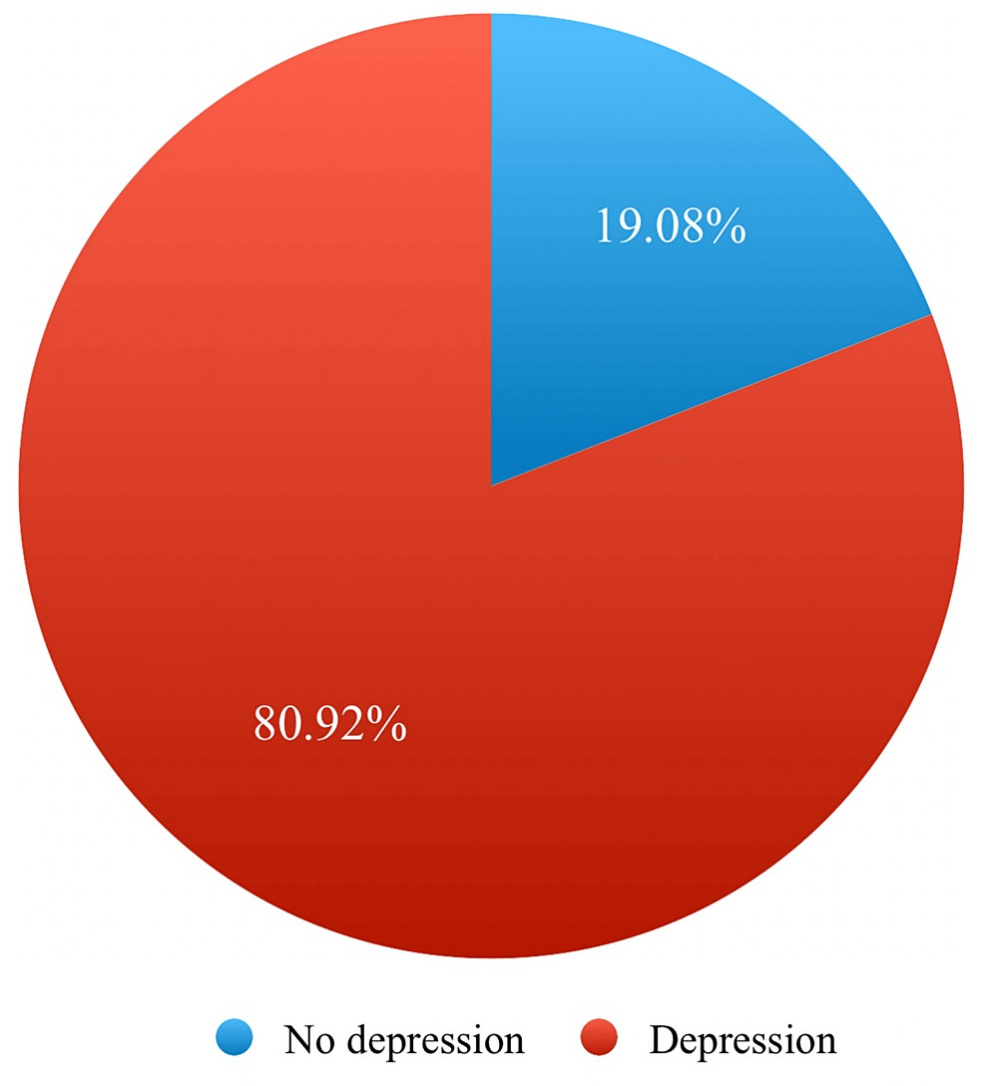
Prevalence of depression among study participants (N = 414).

Based on the results of the PHQ-9 survey, a significant proportion of patients experienced depression after thyroid surgery (Figure 1).

Prevalence of depression severity

**Figure 2:**
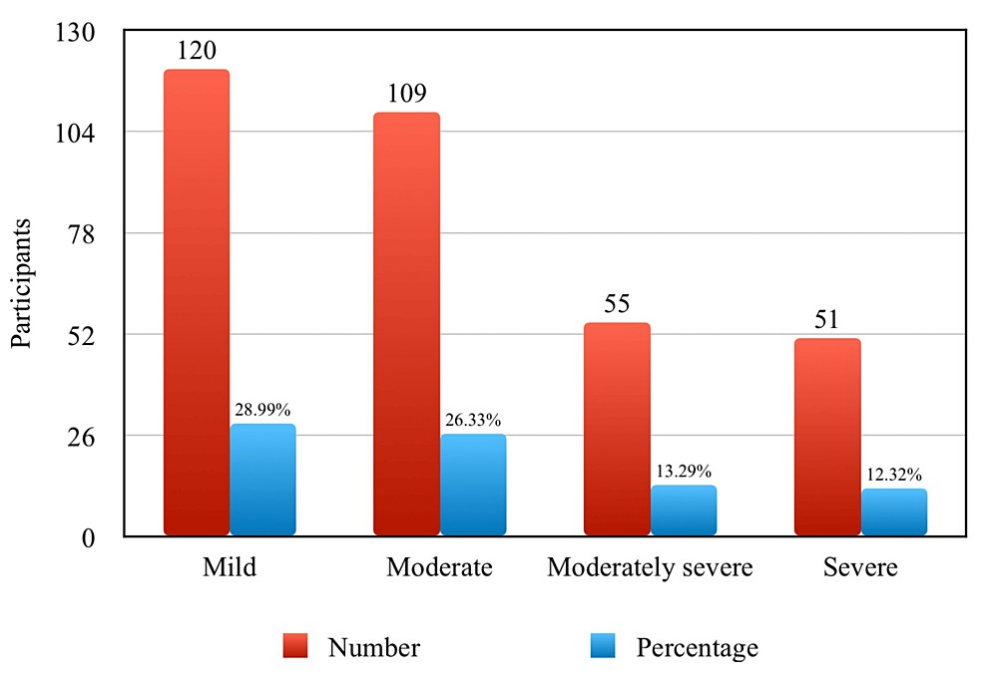
Depression severity among study participants (N = 414).

Most participants suffering from depression had a mild degree (28.99%), while a severe degree of depression was observed in only 12.32% (Figure 2).

Associations between different demographic factors and depression status

Education level was the only factor that showed a significant difference in depression after thyroid surgery. On the other hand, the area of work and a family history of depression showed no significant difference in depression status (Table 3).

**Table 3:** Demographic factors associated with depression after thyroid surgery (N = 414).

Factors	Category	Depression status		P-value
		No depression, n (%)	Depression, n (%)	
Age (year)	19–30	43 (10.39%)	157 (37.92%)	0.493
	31–50	28 (6.76%)	139 (33.57%)	
	Over 50	8 (1.93%)	39 (9.42%)	
Gender	Female	53 (12.80%)	253 (61.11%)	0.426
	Male	26 (6.28%)	82 (19.81%)	
Region	Central region	15 (3.62%)	92 (22.22%)	0.364
	Eastern province	16 (3.86%)	58 (14.00%)	
	Southern area	12 (2.90%)	44 (10.62%)	
	Northern area	5 (1.21%)	29 (7.00%)	
	Western region	31 (7.49%)	112 (27.05%)	
Education	High school education	14 (3.38%)	69 (16.67%)	0.009
	Less than secondary	5 (1.21%)	36 (8.70%)	
	Postgraduate	13 (3.14%)	49 (11.84%)	
	University education	47 (11.35%)	181 (43.72%)	
Area of work	A broker for securities and commodities	3 (0.72%)	7 (1.69%)	0.059
	Environmental management and waste service	1 (0.24%)	1 (0.24%)	
	Legal services	6 (1.45%)	16 (3.86%)	
	Manufacturing or production	1 (0.24%)	10 (2.42%)	
	Organization and associations department	1 (0.24%)	9 (2.17%)	
	Otherwise	57 (13.77%)	233 (46.28%)	
	Personal services		11 (2.65%)	
	Public or private transportation	4 (0.97%)	12 (2.90%)	
	Real estate	3 (0.72%)	18 (4.35%)	
	Social service	3 (0.72%)	18 (4.35%)	
Family history of depression	No	49 (11.84%)	135 (32.61%)	0.325
	Yes	30 (7.25%)	200 (48.31%)	

Associations between different clinical factors and depression status

Surgery time was the only factor that showed a significant difference in depression after thyroid surgery. On the other hand, type of disease, type of surgery, and complications showed no significant difference in depression status (Table 4).

**Table 4:** Clinical factors associated with depression after thyroid surgery (N = 414).

Factors	Category	Depression status		P-value
		No depression, n (%)	Depression, n (%)	
Type of disease	Benign reasons	66 (15.94%)	224 (54.11%)	0.168
	Malignant causes	13 (3.14%)	111 (26.81%)	
Type of surgery	Complete thyroidectomy	40 (9.66%)	177 (42.75%)	0.231
	Excision of a part of the thyroid gland	39 (9.42%)	158 (38.16%)	
Surgery time	4 months ago	18 (4.35%)	39 (9.42%)	0.021
	After 6 months to a year	9 (2.17%)	36 (8.70%)	
	From 4 to 6 months	5 (1.21%)	38 (9.18%)	
	From 1 to 2 years	8 (1.93%)	71 (17.15%)	
	More than 2 years	39 (9.42%)	151 (36.47%)	
Complication	Yes	24 (5.80%)	216 (52.17%)	0.26
	No	55 (13.29%)	119 (28.74%)	
Duration of complications	Ongoing	10 (2.42%)	97 (23.43%)	0.055
	Temporary	16 (3.86%)	147 (35.51%)	
	No complications	53 (12.80%)	91 (21.98%)	

## Discussion

Thyroidectomy is one of the most common procedures worldwide, and one of its consequences is the risk of developing postoperative depression symptoms. A few studies have been conducted to determine the prevalence of post-thyroidectomy depression. One study from South Korea showed a low prevalence, while our study showed a higher prevalence. This could be because we screened patients according to the PHQ-9 and we covered depression status from mild to severe symptoms, while the other study investigated patients for major depressive disorder diagnosed by psychiatrists [11].

Most study participants who underwent thyroidectomy were female and had more severe depression symptoms than males, which may be due to the higher prevalence of thyroid diseases in females than males, similar to this study which showed a higher prevalence of thyroid disease in females [14].

The majority of participants who showed depression symptoms had a higher educational level, which could be explained as having a higher education exposes individuals to more stress and workload leading to depression. In contrast, a cohort study showed that a higher educational level was linked with low depression symptoms [15].

Our study revealed that post-thyroidectomy depression was more common in benign disease, in contrast to another study that found post-thyroidectomy depression was more common in thyroid cancer patients. The fact that our study included both benign and malignant diseases, while the other study only included malignant and a control group could account for the discrepancy [16].

Most participants with depression symptoms had undergone a total thyroidectomy, and, to our knowledge, this can lead to hypothyroidism as thyroid hormone has a significant impact on mental health, unlike in a partial thyroidectomy, where the remaining part mostly compensates for the deficit and has a lower risk of developing depression. Similar findings were noted in a previous study [17].

We found that the prevalence of depression increased gradually after two years of thyroidectomy. Unlike another study that showed the risk reaching up to one year postoperatively, perhaps the distinction between the two studies was that our study was applied to all of our participants who have postoperative depression, whereas the other study assessed a particular group of young adults, females, urban residents, and low-income groups [16].

A substantial number of participants had temporary complications and showed depression. Another previous analytical study showed a higher rate of temporary complications than permanent complications, which could depend on patient factors, surgeon experience, and hospital facilities [18].

Limitations

This was the first study to be conducted in Saudi Arabia and highlights major public health issues such as depression after a common surgery. The study was performed using online questionnaires rather than interviews in hospitals due to the difficulty of hospital access and setup across Saudi Arabia. However, this allowed for the coverage of a larger geographic area. In addition, we also recruited a medium-sized sample as we wanted our data to be as accurate as possible and limited to people who underwent thyroidectomy.

## Conclusions

A significant prevalence of depression was noted among patients undergoing thyroidectomy, which was mostly mild. In addition, most participants were female and had more severe symptoms than males. Furthermore, total thyroidectomy had a higher prevalence of depression than partial thyroidectomy.

The associated factors, such as the educational level, showed more depression symptoms with a high educational level. The risk of developing depression persisted beyond two years after surgery. We recommend regular follow-ups with a psychiatrist for those who underwent thyroidectomy for early detection of depression and timely management.
